# Differential analysis of transcriptomic and metabolomic of free fatty acid rancidity process in oil palm (*Elaeis guineensis*) fruits of different husk types

**DOI:** 10.3389/fpls.2023.1132024

**Published:** 2023-03-08

**Authors:** Shuyan Zhang, Weisheng Zhang, Jerome Jeyakumar John Martin, Rashad Qadri, Xiaopeng Fu, Meili Feng, Lu Wei, Anni Zhang, Cheng Yang, Hongxing Cao

**Affiliations:** ^1^ Coconut Research Institute, Chinese Academy of Tropical Agricultural Sciences / Hainan Key Laboratory of Tropical Oil Crops Biology, Wenchang, China; ^2^ National Key Laboratory for Germplasm Innovation & Utilization of Horticultural Crops, College of Horticulture and Forestry Sciences, Huazhong Agricultural University, Wuhan, China

**Keywords:** oil palm, free fatty acid, rancidity, transcriptome, metabolite

## Abstract

**Introduction:**

Oil palm is the world's highest yielding oil crop and its palm oil has high nutritional value, making it an oilseed plant with important economic value and application prospects. After picking, oil palm fruits exposed to air will gradually become soft and accelerate the process of fatty acid rancidity, which will not only affect their flavor and nutritional value, but also produce substances harmful to the human body. As a result, studying the dynamic change pattern of free fatty acids and important fatty acid metabolism-related regulatory genes during oil palm fatty acid rancidity can provide a theoretical basis for improving palm oil quality and extending its shelf life.

**Methods:**

The fruit of two shell types of oil palm, Pisifera (MP) and Tenera (MT), were used to study the changes of fruit souring at different times points of postharvesting, combined with LC-MS/MS metabolomics and RNA-seq transcriptomics techniques to analyze the dynamic changes of free fatty acids during fruit rancidity, and to find out the key enzyme genes and proteins in the process of free fatty acid synthesis and degradation according to metabolic pathways.

**Results and discussion:**

Metabolomic study revealed that there were 9 different types of free fatty acids at 0 hours of postharvest, 12 different types of free fatty acids at 24 hours of postharvest, and 8 different types of free fatty acids at 36 hours of postharvest. Transcriptomic research revealed substantial changes in gene expression between the three harvest phases of MT and MP. Combined metabolomics and transcriptomics analysis results show that the expression of SDR, FATA, FATB and MFP four key enzyme genes and enzyme proteins in the rancidity of free fatty acids are significantly correlated with Palmitic acid, Stearic acid, Myristic acid and Palmitoleic acid in oil palm fruit. In terms of binding gene expression, the expression of FATA gene and MFP protein in MT and MP was consistent, and both were expressed higher in MP. FATB fluctuates unevenly in MT and MP, with the level of expression growing steadily in MT and decreasing in MP before increasing. The amount of SDR gene expression varies in opposite directions in both shell types. The above findings suggest that these four enzyme genes and enzyme proteins may play an important role in regulating fatty acid rancidity and are the key enzyme genes and enzyme proteins that cause differences in fatty acid rancidity between MT and MP and other fruit shell types. Additionally, differential metabolite and differentially expressed genes were present in the three postharvest times of MT and MP fruits, with the difference occurring 24 hours postharvest being the most notable. As a result, 24 hours postharvest revealed the most obvious difference in fatty acid tranquility between MT and MP shell types of oil palm. The results from this study offer a theoretical underpinning for the gene mining of fatty acid rancidity of various oil palm fruit shell types and the enhancement of oilseed palm acid-resistant germplasm cultivation using molecular biology methods.

## 1 Introduction

Oil palm (*Elaeis guineensis*) is the most efficient oilseed crop in the world, with an average annual oil yield of 4270 kg/hm^2^, which is 7-8 times higher than peanut and 9-10 times higher than soybean per unit area and is known as the “world oil king” (Murphy, 2007; Xia et al., 2011; Mba et al., 2015) and has rich economic value (Xiong et al., 2009; Damayant et al., 2014). The fruit is the oil-producing organ of oil palm and the vegetable oil extracted from the mesocarp and kernel is known as palm oil (PO) and palm kernel oil (PKO), respectively (Dussert et al., 2013).The mesocarp and the kernel of oil palm make this crop unique and economically important due to their extraordinarily high oil contents (approximately 85% and 50% dry mass, respectively). PO has excellent characteristics such as high nutritional and oxidative stability (Olafisoye et al., 2015; Kadandale et al., 2019). It is low in sterols, rich in vitamin A and vitamin E, and contains 50% saturated fatty acids, 40% monounsaturated fatty acids, and 10% polyunsaturated fatty acids (Clegg, 1973; Manorama and Rukmini, 1991; Boateng et al., 2016).

Free fatty acids, also known as non-esterified fatty acids, are important substrates for cell energy metabolism and provide energy for the heart, liver, skeletal muscle and other organs. An imbalance of free fatty acid metabolism in plasma can lead to coronary atherosclerosis, diabetes and other diseases (Liu, 2021). Therefore, it is important to study free fatty acids in PO. Free fatty acids are susceptible to light, heat, oxygen and moisture in the air and enzymes break down under the influence of such factors as the oxide and hydrogen peroxide, and the intermediates that are then decomposed to aldehydes, ketones,and other compounds that lose their nutritional value and other values, this kind of phenomenon is called rancidity of fatty acids of deterioration (Yang et al., 2010). According to the different ways of producing hydroperoxides during fatty acid oxidation, the oxidation can be roughly divided into three categories: automatic oxidation, photooxidation and enzymatic oxidation (Li et al., 1997; Sun et al., 1998; Ahmed et al., 2016). Automatic oxidation is the reaction of fatty acids directly with oxygen in the air at room temperature. The reaction is completely spontaneous without direct light irradiation or the artificial addition of any catalyst. The primary mode of deterioration for vegetable oils is automatic oxidation (Wang, 2004). The oxidized rancidity of oil not only affects the flavor, color and nutritional quality of foods, but also produces toxic substances such as peroxides that are harmful to the human body in the process of rancidity, which affects the health of consumers (Wang et al., 2014). In severe cases, it can accelerate aging and cause diseases such as tumors and cardiovascular disease. Therefore, it is important to have a thorough understanding of the factors that affect lipid oxidation, which helps us take effective measures to inhibit oil oxidation and extend the shelf life. Fatty acyl-ACP thioesterase (*FAT*) is a key enzyme that regulates the elongation of fatty acid carbon chains during fatty acid synthesis. It can catalyze the hydrolysis of thioester bonds to terminate fatty acid synthesis, and its activity affects the content of various fatty acids in plants (Mayer and Shanklin, 2007). The Short chain dehydrogenase (Short-chain dehydrogenases/reductase, *SDR*) is the most widely distributed kind of oxidoreductase, in plant metabolism and plays an important role in the process (Gong et al., 2020). The fatty acid β-oxidation multifunctional protein (*MFP*) is a microsomal enzyme that catalyzes the hydration and dehydrogenation processes of the -oxidation pathway, which are accompanied by energy production (Novikov et al., 1994; Dieuaide-Noubhani et al., 1996; Dieuaide-Noubhani et al., 1997).

The fruit of the oil palm produces PO, which has a lower oil content in immature fruits than in mature fruits but a higher content of free fatty acids and an increased risk of rancidity in overripe fruits. Exposure to the air gradually softens the fruit, accelerating the rancidity of fatty acids. As a result, oil palm should be processed shortly after harvesting in order to maintain oil quality. In this study, the fruits of two oil palm shell types, MT and MP, were selected to analyze the dynamic changes in free fatty acid metabolite content and differentially expressed genes at 0 h, 24 h and 36 h after harvesting, combined with LC-MS/MS metabolomics technology and RNA-Seq transcriptomics technology. This study provides a theoretical basis for gene mining of fatty acid rancidity in different shell types of oil palm, improvement of fatty acid composition of palm oil and palm kernel oil, gene editing and breeding of new varieties.

## 2 Materials and methods

### 2.1 Plant materials

Fresh oil palm (*Elaeis guineensis*) fruits were harvested at the National Tropical Palm Germplasm Resource Nursery, Wenchang, Hainan Province, China (110.8°latitude, 19.6°longitude) 185 days after pollination from Pisifera (MP) and Tenera (MT) plants. After collection samples were left at room temperature for 0 h, 24 h, and 36 h. For further examination, samples were then immediately flash-frozen in liquid nitrogen and kept at -80°C. MP1, MP2 and MP3 were recorded at 0 h, 24 h and 36 h rancidity under natural conditions 185 days after MP pollination. The fruits of rancidity at 0 h, 24 h and 36 h under natural conditions 185 days after MT pollination were denoted as MT1, MT2 and MT3, respectively. MP and MT samples were taken for 3 replicates.

### 2.2 Methods

#### 2.2.1 Metabolite extraction

For the metabolite determination, 20 mg of frozen sample was placed in a centrifuge tube, 1 mL of lipid extract (methyl tert-butyl ether: methanol =3:1, V/V, including internal standard mixture), and the tube was then closed and shaken for 30 minutes. After adding 300μL of ultrapure water, the tube was shaken for 1 minute before being placed at 4°C for 10 minutes. Then the tube was centrifuged for 3 minutes (12000 rpm, 4°C) and 400 μL of supernatant was transferred into a new centrifuge tube and concentrated at 20°C for 2 hours, or until completely dry. The solution was then vortexed for 3 minutes and centrifuged for 10 minutes (12000 rpm, 4°C) with 200 μL of lipid complex solution (acetonitrile: isopropanol =1:1, V/V). Following that, 120 μL of supernatant was transferred into the glass-lined tube until it was used.

#### 2.2.2 Metabolomics analysis and data processing

The instrument platform for LC-MS/MS analysis was composed of ExionLC ultra-performance liquid chromatography in tandem with SCIEX QTRAP 6500+ mass spectrometer, and the chromatographic column was Thermo AccucoreC30 column (2.6 μm, 2.1 mm×100 mm i.d.). The mobile phases were: Phase A, acetonitrile/water (60/40, V/V) (containing 0.1% formic acid, 10 mmol/L ammonium formate); Phase B, acetonitrile/isopropanol (10/90, V/V) (containing 0.1% formic acid, 10 mmol/L ammonium formate). Gradient elution method: At 0 minutes, the A/B ratio was 80:20 (V/V), 70:30 (V/V) at 2 minutes, 40:60 (V/V) at 4 minutes, 15:85 (V/V) at 9 minutes, 10:90 (V/V) at 14 minutes, 5:95 (V/V) at 15.5 minutes. 5:95(V/V) at 17.3 minutes, 80:20(V/V) at 17.5 minutes, and 80:20(V/V) at 20 minutes. The flow rate was 0.35 ml/min, the column temperature was 45°C, and the injection volume was 2 μL. The main conditions for tandem mass spectrometry were Electrospray Ionization (ESI) temperature 500 °C, mass spectrum voltage 5500 V in positive ion mode, and -4500 V in negative ion mode. In the triple quadrupole, ion source gas 1 (GS1), gas 2 (GS2), and curtain gas (CUR) are at 45 psi, 55 psi, and 35 psi, respectively. Each ion pair is based on the optimized declustering potential (DP) and collision energy (CE) used for scanning detection.Three replicates of oil palm fruits in each treatment time were taken in parallel.

The software Analyst 1.6.3 was used to process the mass spectral data and combined with the information of the local lipid database, mass spectral qualitative analysis was performed on the metabolites of the samples. The mass spectral peaks detected for the same metabolite in different samples were corrected, and the integrated peak areas of all samples detected were calculated to obtain the absolute content of metabolites. The MRM model results are shown in **Supplementary Figure 1**. Principal component analysis (PCA) and orthogonal partial least squares analysis (OPLS-DA) were performed on 18 oil palm fruit samples from three treatments. OPLS-DA screening model Variable VIP(Variable Importance in the Pmiectio)≥1, and a combination of Fold-Change method to screening differential metabolite, with screening criteria Fold_Change≥2 and Fold_Change ≤ 0.5.

#### 2.2.3 Total RNA extraction and high-throughput sequencing

Total RNA was extracted from 18 samples of oil palm MP and MT fruits using the instructions of RNA extraction kit. After obtaining RNA, the integrity of RNA was analyzed by agarose gel electrophoresis. The RNA purity (OD260/280 and OD260/230 ratio) was detected by NanoPhotometer spectrophotometer. The Qubit 2.0 Fluorometer was used to accurately measure RNA concentration. The Agilent 2100 bioanalyzer was used to accurately detect RNA integrity.

Total RNA(≥1ug) was used for library construction. Illumina’s NEBNext^®^UltraTM RNA Library Prep Kit was used for library construction. The mRNA with polyA tail was enriched by Oligo(dT) magnetic beads, which were then randomly interrupted by divalent cations in NEB Fragmentation Buffer. The first strand of cDNA was synthesized in the M-MuLV reverse transcriptase system using fragmented mRNA as a template and random oligonucleotides as primer. The RNA strand was then degraded by RNaseH and the second strand was synthesized by dNTPs in the DNA polymerase I system. After the end repair, A-tail was added and sequencing adaptor was connected, AMPure XP beads were used to screen about 200bp of cDNA, PCR amplification was performed, and AMPure XP beads were used to purify PCR products. Finally, the library was obtained. After library construction, the library was initially quantified using Qubit2.0 Fluorometer, diluted to 1.5ng/ul, and then the insert size of the library was detected using Agilent 2100 bioanalyzer. qRT-PCR was used to accurately quantify the effective concentration of the library (library effective concentration higher than 2nM) to ensure the quality of the library. After passing the library inspection, different libraries were pooled according to the requirements of effective concentration and target off-machine data volume for Illumina sequencing, and 150bp paired-end readings were generated. The basic principle of Sequencing is Sequencing by Synthesis. In the sequencing of the flow cell with four kinds of fluorescence-labeled dNTP, primers for amplification, DNA polymerase and joint in each sequence complementary chain cluster, each fluorescently labeled dNTP can release the corresponding fluorescence, and the sequencing machine captures the fluorescent signal. Through the computer software to convert optical signals into sequencing peaks, the sequence information of the fragment to be tested is obtained.

#### 2.2.4 Transcriptome data processing and differentially expressed genes analysis

Fastp v 0.19.3 was used to filter the original data, mainly removing reads with adapter. When the N content of any measured reads exceeds 10% of the base number of reads, paired reads are removed. When the number of low-quality (Q ≤ 20) bases in any sequencing reads exceeds 50% of the number of bases in the reads, paired reads are removed. Clean reads were obtained for all subsequent analysis. HISAT v2.1.0 was used to construct the index, and the clean reads alignment sequence was aligned to the downloaded reference genome and data file. Novel gene prediction was performed using StringTie v1.3.4d, and the network flow algorithm and optional *de novo* assembly (*de novo*) were applied to splice transcripts. The recounts were calculated using featureCounts v1.6.2, and the FPKM of each gene was calculated with the recount length. Differential expression analysis between the two groups was performed using DESeq2 v1.22.1, with P-value corrected using the Benjamini&Hochberg method. Adjusted P-value and | log2Fold_Change | as the thresholds of significant differences in expression, gene screening conditions for | log2Fold_Change | ≥1 or higher, and FDR < 0.05. The quality of RNA-seq is shown in **Supplementary Table 1**.

## 3 Results

### 3.1 Metabolomic analysis of lipids and free fatty acids

**Figure 1:**
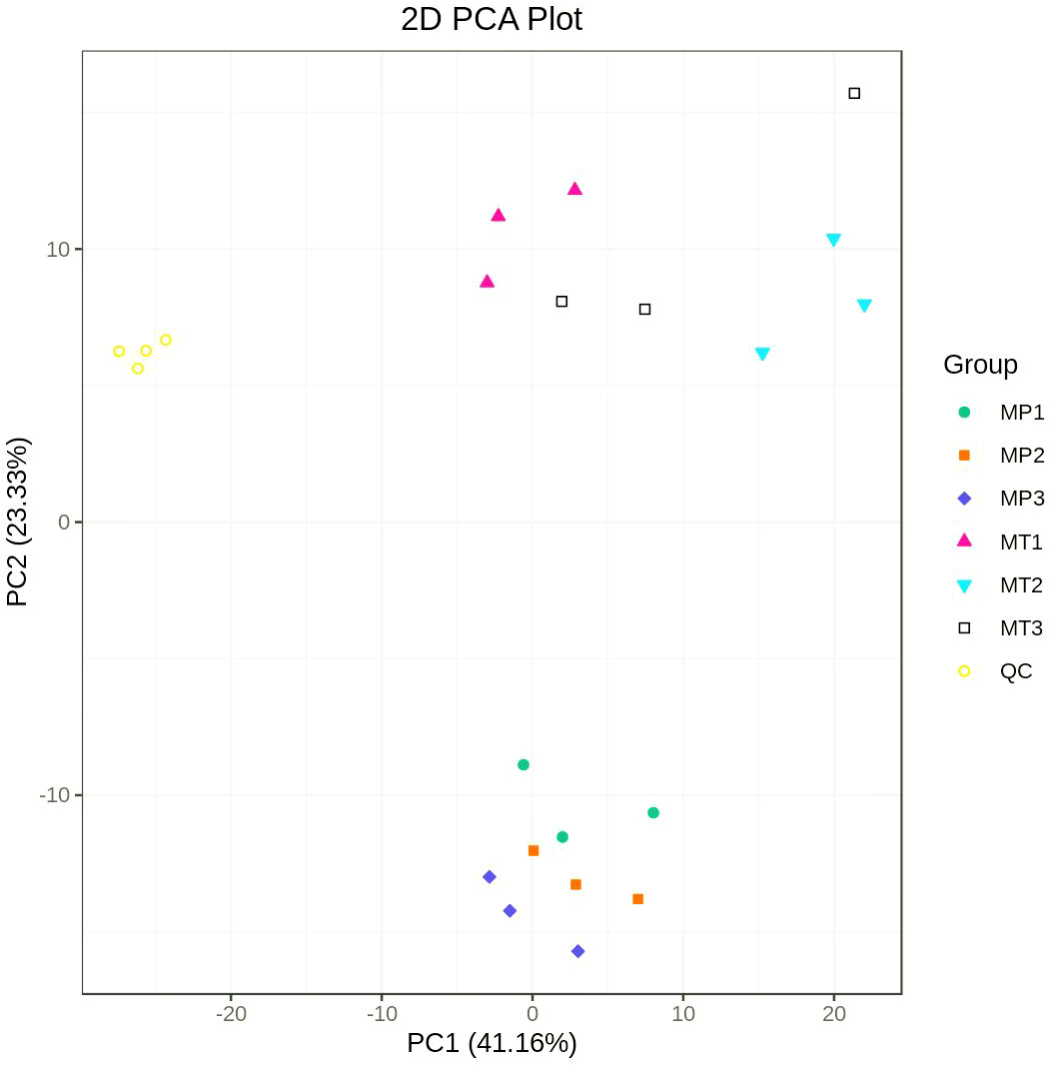
Principal component analysis of MP and MT overall samples. MP1 (MT1): 0h postharvest fruit under natural conditions; MP2 (MT2): 24h postharvest fruit under natural conditions; MP3 (MT3): 36h postharvest fruit under natural conditions; QC is the quality control sample.

Principal component analysis (PCA) was performed on the composition of different lipid metabolites (including quality control samples) of MP and MT oil palm fruits at 0 h, 24 h and 36 h postharvest times. **Figure 1** shows that the separation degree of MP and MT sample group data is clear, and there is a good distinction between the groups. The first principal component (PC1) and the second principal component accounted for 41.16% and 23.33% of the total variables respectively.

The lipid differential metabolite of oil palm MP and MT fruits in different postharvest times were screened using orthogonal partial least squares discriminant analysis (OPLS-DA) with VIP = 1, Fold Change ≥ 2, and Fold Change ≤ 0.5, and the significant differential metabolite were statistically and compared. **Supplementary Figure 2A** shows that there are 99 differential metabolite were detected between MP1-vs-MT1, 120 differential metabolite were detected between MP2-vs-MT2, and 138 differential metabolite were detected between MP3-vs-MT3. **Supplementary Figure 2B** shows that there are 63 up-regulated and 36 down-regulated differential metabolites between MP1-vs-MT1 and 102 up-regulated and 18 down-regulated differential metabolites between MP2-vs-MT2, but 117 up-regulated and 21 down-regulated differential metabolites were detected between MP3-vs-MT3. These findings indicated that the lipid differential metabolite of MP and MT shell types increased with the increase in postharvest time, as did the number of up-regulated metabolites.

**Figure 2:**
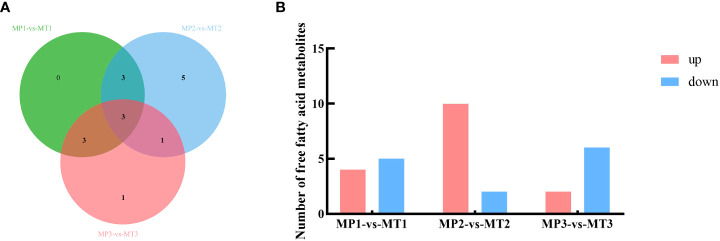
Statistics of MP and MT oil palm significant differential metabolite **(A)** Venn diagram of metabolites with a significant difference in free fatty acids; **(B)** Histogram of free fatty acid metabolism with a significant difference.

The differential metabolite related to the rancidation of free fatty acids in MP and MT fruits of oil palm at different postharvest times was also screened by the above method. As shown in **Figure 2A**, 9 differential metabolites were detected between MP1-vs-MT1, 12 differential metabolites were detected between MP2-vs-MT2, and 8 differential metabolites were detected between MP3-vs-MT3. **Figure 2B** shows that there are 4 up-regulation and 5 down-regulation differential metabolites between MP1-vs-MT1 and 10 up-regulation and 2 down-regulation differential metabolites between MP2-vs-MT2, however 2 up-regulation and 6 down-regulation differential metabolites were detected between MP3-vs-MT3. These results indicated that MP2-vs-MT2 had the most significant difference in rancidity between the two shell types.

The changes in free fatty acid content of oil palm at three different postharvest times are shown in **Table 1**. Among them, the content of saturated fatty acid palmitic acid was the highest in both MP and MT, but the content of MT was significantly higher than that of MP. In addition, the content changes with different postharvest time points in MP; it first decreases and then increases, reaching its highest in MP3. However, in MT, its content first increased and reached its highest content in MT2 and then decreased. Second, the content of steric acid, which is a type of saturated fatty acid found in oil palm, was significantly higher in MT than in MP. The contents of oleic acid and linoleic acid were the most unsaturated fatty acids, and the contents of these two fatty acids showed different trends in MP and MT. The contents of oleic acid decreased continuously in the MP1-MP3 stages, while the contents of oleic acid increased first and then decreased in MT1-MT3 stages. In the MP1-MP3 stages, the content of linoleic acid decreased first and then increased, and reaching its highest content in the MP3 stage, while in the MT1-MT3 stages, the content change trend was the same as that of oleic acid.

**Table 1:** Changes of free fatty acids in MP and MT rancidity at different stages.

Fatty acid	Changes of free fatty acids in MP and MT rancidity at 0h, 24h and 36h postharvest (nmol/g)					
	MP1	MP2	MP3	MT1	MT2	MT3
C10:0 Decanoic acid	206.91	235.79	181.36	239.25	212.12	192.70
C12:0 Lauric acid	8.91	14.69	27.72	7.54	16.81	17.66
C14:0 Myristic acid	101.73	74.94	256.81	371.80	553.29	581.39
C15:0 Pentadecanoic acid	36.51	33.05	104.44	40.47	58.84	54.60
C16:0 Palmitic acid	8 060.21	7 844.82	12 946.03	14 345.15	17 834.69	16 177.21
C16:1 Palmitoleic acid	247.14	153.08	615.26	118.02	162.31	151.12
C17:0 Heptadecanoic acid	103.22	98.20	282.87	137.42	228.11	203.24
C17:1 Heptadecenoic acid	125.01	89.95	264.70	41.74	71.82	55.26
C18:0 Stearic acid	1 756.83	1 579.47	3 270.60	2 988.75	4 480.60	3 807.98
C18:1 Oleic acid	32 992.80	29 659.44	28 690.32	30 396.86	31 704.01	26 683.76
C18:2 Linoleic acid	13 475.84	10 080.90	17 552.40	10 188.00	15 804.62	11 046.89
C18:3 Linolenic acid	237.34	172.55	560.86	189.08	431.63	281.12
C19:1 Nonadecenoic acid	29.67	21.72	71.95	12.10	21.03	15.75
C20:0 Arachidic acid	75.33	77.28	330.68	178.70	403.69	299.03
C20:1 Eicosaenoic acid	395.73	300.02	726.46	392.81	570.14	472.84
C20:2 Eicosadienoic acid	3.67	2.49	10.14	3.96	6.77	5.33
C22:0 Behenic acid	42.29	51.82	134.03	104.06	223.00	164.21
C22:3 Docosatrienoic acid	19.44	16.87	23.20	22.56	25.13	22.53
C22:4 Docosatetraenoic acid	5.60	5.07	7.29	6.14	6.25	6.07
C22:6 Docosahexaenoic acid	9.55	8.18	31.49	7.15	9.35	9.59
C24:0 Lignoceric acid	63.93	68.41	209.22	128.63	298.43	222.01
C24:5 Docosapentaenoic acid	9.76	7.72	8.19	10.66	10.47	9.10
C24:6 Nisioic acid	45.71	42.61	43.52	55.90	59.22	48.98
C28:0 Octacosanoic acid	78.27	57.06	59.38	102.38	137.54	125.64
C30:0 Tricosanoic acid	11.78	5.40	6.22	3.52	2.28	2.43
C32:0 Dotriacontanoic acid	7.80	4.41	3.72	2.13	2.00	1.75
C33:0 Tristridecanoic acid	9.23	9.12	6.48	6.87	6.98	6.39
C34:0 Gheddic acid	7.88	8.37	5.58	7.68	7.25	4.86
C35:0 Tripentacosanoic acid	33.83	31.87	23.63	34.80	30.81	24.65
C36:0 Trihexadecanoic acid	29.48	27.66	20.57	30.63	28.19	21.59
Total fatty acid content	58 231.4	50 782.96	66 475.12	60 174.76	73 407.38	60 715.68

At the same postharvest time points, the fatty acid differential metabolite in MP and MT were compared and the results are shown in **Table 2**. The fatty acid results revealed significant differences between MP1-vs-MT1 (in Arachidic acid, Palmitoleic acid, Heptadecenoic acid, Nonadecenoic acid, Dotriacontanoic acid Tricosanoic Lignoceric acid, Myristic acid and Behenic acid) in, MP2-vs-MT2 in the fatty acids of (in Arachidic acid, Linolenic acid, Eicosadienoic acid, Dotriacontanoic acid, Stearic acid Acid, Tricosanoic acid, Octacosanoic acid, Lignoceric acid, Myristic acid, Palmitic acid, Heptadecanoic acid) and Behenic acid, MP3-vs-MT3 (in Palmitoleic acid, Heptadecenoic acid, Nonadecenoic acid, Dotriacontanoic acid, Tricosanoic acid, Octacosanoic Myristic acid, and Docosahexaenoic acid). However, the fatty acids Dotriacontanoic acid, Tricosanoic acid and Myristic acid were found to be different in between the three groups.

**Table 2:** Statistics of significant differential metabolite of free fatty acids between groups of MP and MT rancidity in different times.

Fatty acid	VIP/Fold-Change		
	MP1-vs-MT1	MP2-vs-MT2	MP3-vs-MT3
C20:0 Arachidic acid	**1.28/2.37**	**1.30/5.22**	0.77/0.90
C16:1 Palmitoleic acid	**1.27/0.48**	0.74/1.06	**1.32/0.25**
C17:1 Heptadecenoic acid	**1.30/0.33**	1.08/0.80	**1.32/0.21**
C18:1 Oleic acid	0.63/0.92	0.71/1.07	0.78/0.93
C19:1 Nonadecenoic acid	**1.27/0.41**	0.28/0.97	**1.32/0.22**
C18:3 Linolenic acid	1.17/0.80	**1.29/2.50**	1.21/0.50
C18:2 Linoleic acid	1.17/0.76	1.28/1.57	1.30/0.63
C20:2 Eicosadienoic acid	0.46/1.08	**1.27/2.72**	1.27/0.53
C36:0 Trihexadecanoic acid	0.24/1.04	0.17/1.02	0.65/1.05
C22:3 Docosatrienoic acid	0.84/1.06	1.20/1.49	0.39/0.97
C22:4 Docosatetraenoic acid	0.52/1.10	0.90/1.23	1.01/0.83
C20:1 Eicosenoic acid	0.10/1.00	1.28/1.90	1.26/0.65
C35:0 Tripentacosanoic acid	0.21/1.03	0.23/0.97	0.65/1.04
C32:0 Dotriacontanoic acid	**1.30/0.27**	**1.21/0.45**	**1.10/0.47**
C33:0 Tristridecanoic acid	1.13/0.75	1.06/0.77	0.14/0.99
C18:0 Stearic acid	1.30/1.70	**1.28/2.84**	0.91/1.16
C30:0 Triaconoic acid	**1.29/0.30**	**1.18/0.42**	**1.26/0.39**
C28:0 Octacosanoic acid	1.06/1.31	**1.27/2.41**	**1.25/2.12**
C24:0 Lignoceric acid	**1.27/2.01**	**1.29/4.36**	0.28/1.06
C12:0 Lauric acid	1.00/0.85	0.77/1.14	1.26/0.64
C14:0 Myristic acid	**1.31/3.65**	**1.30/7.38**	**1.30/2.26**
C22:6 Docosahexaenoic acid	1.22/0.75	1.08/1.14	**1.32/0.30**
C15:0 Pentadecanoic acid	0.58/1.11	1.26/1.78	1.32/0.52
C16:0 Palmitic acid	1.26/1.78	**1.29/2.27**	1.27/1.25
C17:0 Heptadecanoic acid	1.24/1.33	**1.28/2.32**	1.17/0.72
C22:0 Behenic acid	**1.30/2.46**	**1.29/4.30**	0.91/1.23
C34:0 Gheddic acid	0.11/0.97	0.75/0.87	0.66/0.87
C24:6 Nisioic acid	0.93/1.22	1.23/1.39	1.03/1.13
C24:5 Docosapentaenoic acid	0.66/1.09	1.00/1.36	0.82/1.11
C10:0 Decanoic acid	0.69/1.16	0.58/0.90	0.34/1.06

Bold represents a significant difference.

**Figure 3:**
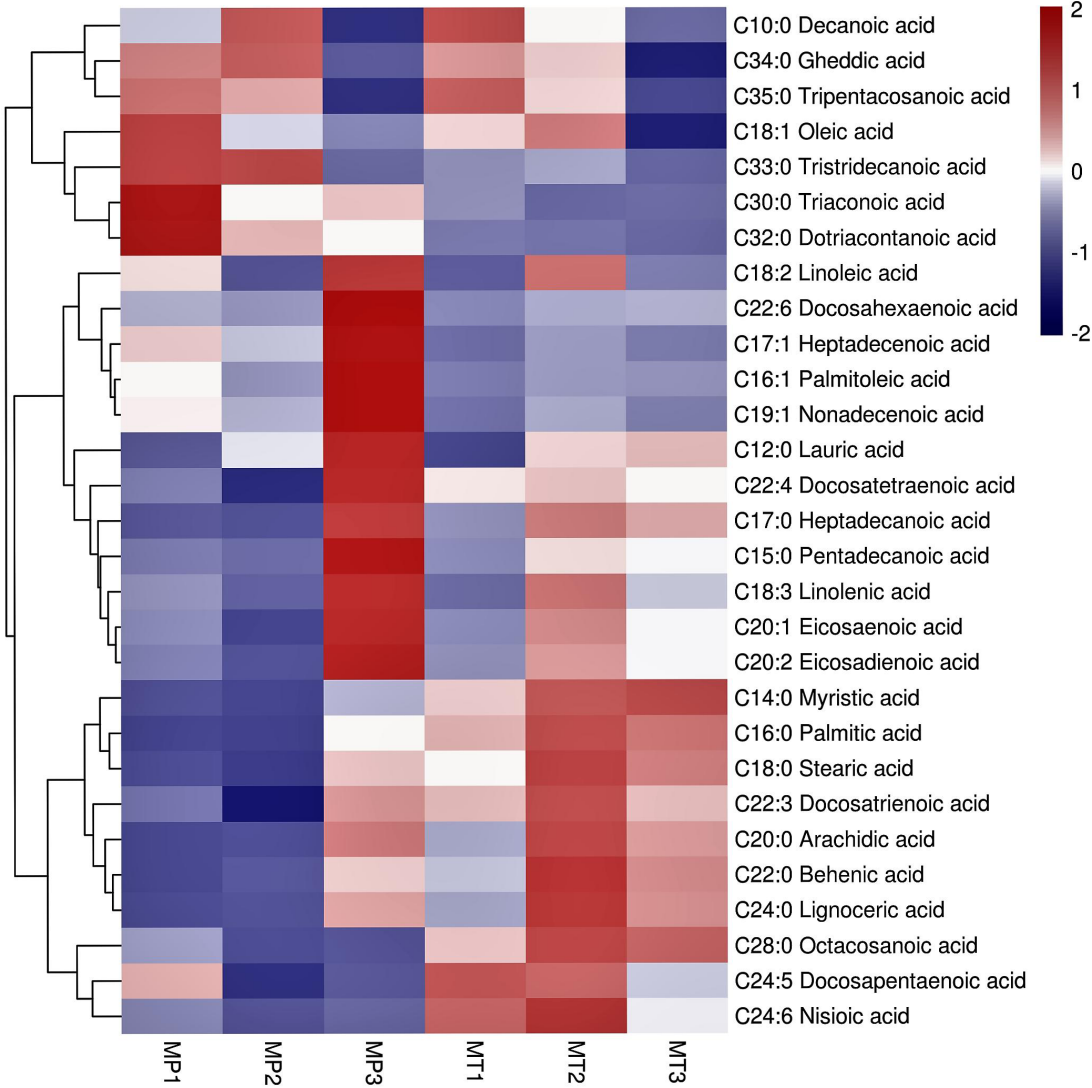
Cluster heatmap of significant differential metabolite for free fatty acids of each group. The 6 columns in each heatmap represent the fruits postharvest stages (MP1, MP2, MP3, MT1, MT2, and MT3).

Cluster analysis of all free fatty acids in MP and MT of the three postharvest times (**Figure 3**) showed that the 29 free fatty acids normalized by Z-score were clustered into two categories. The content of 7 free fatty acids in the first group was higher in the three stages of MP.Among them, the contents of Tripentacosanoic acid, Oleic acid, Tristridecanoic acid, Tricosanoic acid and Dotriacontanoic acid are the highest at MP1 Whereas, the contents of Decanoic acid and Gheddic acid were the highest at MP2. However, the contents of 7 free fatty acids, particularly tricosanoic acid, dotriacontanoic acid, and tristridecanoic acid, were low in the three MT times. In the second category, the content changes of 22 free fatty acids in MP and MT can be divided into two sub-categories. In the first subcategory, the content of 12 free fatty acids was very low in MP1 and MP2 stages, and the content reached its highest in MP3 stage, which was significantly different from that in MP1 and MP2 stages. In MT1-3 stage, the content of free fatty acids in MT1 stage was generally low, but the content in MT2 and MT3 stage was increased, which is significantly different from that in MT1 stage.In the second subcategory, 10 free fatty acids generally have low content in MP and little change. However, the content in MT is very high, among which Myristic acid has the highest content in MT3, Docosapentaenoic acid has the highest content in MT1, and the other 8 free fatty acids have the highest content in the MT2.

In general, the content of free fatty acids of the first category was higher in MP oil palm than in MT. The contents of free fatty acids of the second major group fluctuated, and the above results showed that the changes of free fatty acids of the two shell types were significantly different.

### 3.2 Global analysis of gene expression in different postharvest stages

**Figure 4:**
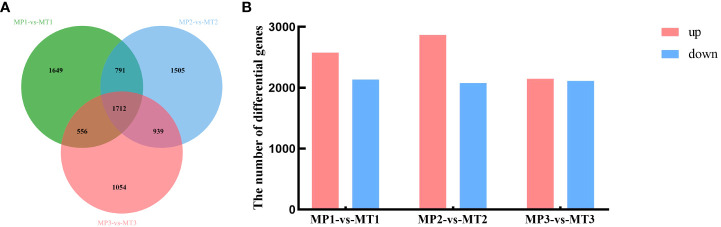
Oil palm differentially expressed genes statistics of MP and MT. **(A)** Venn diagram of differentially expressed genes; **(B)** Histogram of significantly differentially expressed genes.

Significant false discovery rates (FDR) of< 0.05 and | log2Fold_Change | ≥1 was calculated for the MP and MT groups, and significant differentially expressed genes were examined. As shown in **Figure 4A**, 4708 differentially expressed genes were detected between MP1-vs-MT1, 4947 differentially expressed genes between MP2-vs-MT2, and 4261 differentially expressed genes between MP3-vs-MT3. **Figure 4B** showed that 2574 differentially expressed genes were up-regulated and 2134 differentially expressed genes were down-regulated between MP1-vs-MT1. There were 2868 up-regulated differentially expressed genes and 2079 down-regulated differentially expressed genes between MP2-vs-MT2. There were 2150 up-regulated differentially expressed genes and 2111 down-regulated differentially expressed genes between MP3-vs-MT3. The above results showed that MP2-vs-MT2 had the most differentially expressed genes, and the proportion of up-regulated differentially expressed genes was the largest.

### 3.3 Enrichment analysis of significantly differentially expressed genes

**Figure 5:**
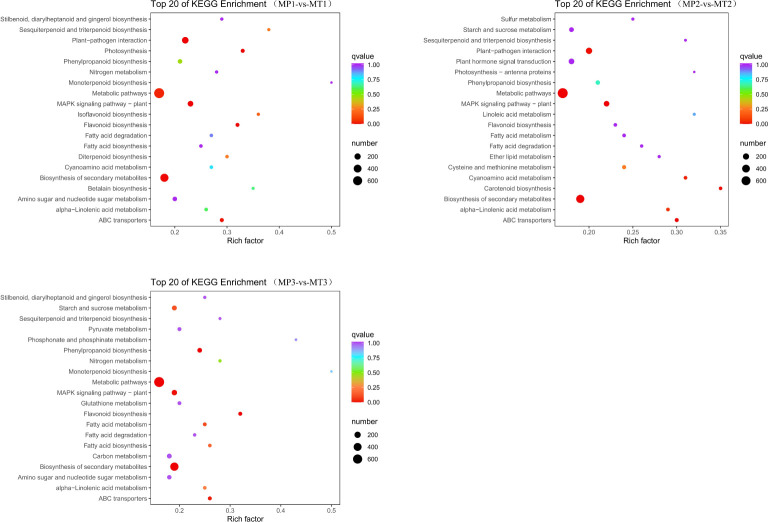
KEGG enrichment classification scatter chart for the pairwise comparisons of MP1-vs-MT1, MP2-vs-MT2, and MP3-vs-MT3.

KEGG metabolic pathway analysis showed that the MP1 vs MT1 comparative group was enriched to 134 pathways; The MP2 vs MT2 comparative group enriched 133 pathways; The MP3 vs MT3 comparative group enriched 133 pathways. As shown in **Figure 5**, the gene enrichment pathways annotated by the three comparison groups are mainly “metabolic pathways”, “biosynthensis of secondary metabolites” and “plant-pathogen interaction”. Based on the above analysis, we further study the specific functional genomes related to free fatty acids.

### 3.4 Combined metabolomics and transcriptomic analysis of fatty acid rancidity

Fatty acid metabolome and transcriptome data were analyzed jointly according to KEGG metabolic pathways, and KEGG pathways enriched by both omics were found according to different postharvest times (**Table 3**). MP1-vs-MT1 was enriched on KEGG into two pathways, Fatty acid biosynthesis and Biosynthesis of unsaturated fatty acids. Palmitoleic acid and Myristic acid were included in fatty acid biosynthesis, and 22 differential genes of this pathway were annotated. The biosynthesis of unsaturated fatty acids pathway contained Arachidic acid, Lignoceric acid, and Behenic acid, and eight differential genes were annotated in this pathway. MP2-vs-MT2 was found to be enriched in five KEGG channels: Fatty acid biosynthesis, Fatty acid metabolism, Fatty acid degradation, Biosynthesis of unsaturated fatty acids, and Fatty acid elongation. Palmitic acid was found in the Fatty acid metabolism pathway, and 29 differential genes were annotated. Palmitic acid was contained in Fatty acid degradation pathways, and 19 differential genes were annotated. This pathway contained Stearic acid, Myristic acid, Palmitic acid, and 20 differential genes were annotated in this pathway. The Biosynthesis of unsaturated fatty acids pathway contained Arachidic acid, Eicosadienoic acid, Stearic acid, Lignoceric acid, Palmitic acid and Behenic acid, 8 differentially expressed genes in this pathway were annotated. Palmitic acid was found in Fatty acid elongation pathway, and eight differentially expressed genes were identified. MP3-vs-MT3 is enriched on KEGG into two pathways, the Fatty acid biosynthesis and the Biosynthesis of unsaturated fatty acids pathway. The Fatty acid biosynthesis pathway contained Myristic acid and Palmitoleic acid, and 23 differential genes were annotated. The pathway in Biosynthesis of unsaturated fatty acids contained Docosahexaenoic acid, and 8 differential genes were annotated.

**Table 3:** KEGG statistics of MT and MP oil palm fatty acid metabolism pathways.

KEGG pathway	ko-id	Cluster- Frequency(%)	Genome- Frequency(%)	P-value	Corrected-P-value
MP1-vs-MT1					
Fatty acid biosynthesis	ko00061	22(1.32%)	88 (0.81%)	0.012141483	1
Biosynthesis of unsaturated fatty acids	ko01040	8 (0.48%)	36 (0.33%)	0.177162487	1
MP2-vs-MT2					
Fatty acid biosynthesis	ko00061	20 (1.20%)	88 (0.81%)	0.042127	1
Fatty acid metabolism	ko01212	29 (1.74%)	122 (1.12%)	0.009149	1
Fatty acid degradation	ko00071	19 (1.14%)	74 (0.68%)	0.014117	1
Biosynthesis of unsaturated fatty acids	ko01040	8 (0.48%)	36 (0.33%)	0.176324	1
Fatty acid elongation	ko00062	8 (0.48%)	52 (0.48%)	0.554664	1
MP3-vs-MT3					
Fatty acid biosynthesis	ko00061	23 (1.56%)	88 (0.81%)	0.001178	0.156674
Biosynthesis of unsaturated fatty acids	ko01040	8 (0.54%)	36 (0.33%)	0.104823	1

The integral values of genes and metabolites were used for correlation analysis, and the cor function in R software was used to calculate the Pearson correlation coefficient of genes and metabolites. The differentially expressed genes and differential metabolite correlations were selected from the overall correlation results for joint analysis. Four fatty acids and four key enzyme genes and proteins related to them were screened out and annotated with NR (**Table 4**).

**Table 4:** Correlation analysis of key enzyme gene expression and main fatty acid content.

Gene ID	Enzyme name	Myristic acid FFA(14:0)	Stearic acid FFA(18:0)	Palmitic acid FFA(16:0)	Palmitoleic acid FFA(16:1)
*LOC105047881*	Palmitoyl-acyl carrier protein thioesterase, chloroplastic (*FATB*)	0.98	0.92	0.95	-0.32
*LOC105035520*	Short-chain type dehydrogenase/reductase-like (*SDR*)	-0.89	-0.81	-0.86	0.51
*LOC105048939*	Oleoyl-acyl carrier protein thioesterase, chloroplastic (*FATA*)	-0.86	-0.78	-0.83	0.54
*LOC105040837*	Peroxisomal fatty acid beta-oxidation multifunctional protein-like (*MFP*)	-0.86	-0.83	-0.84	0.36

**Figure 6:**
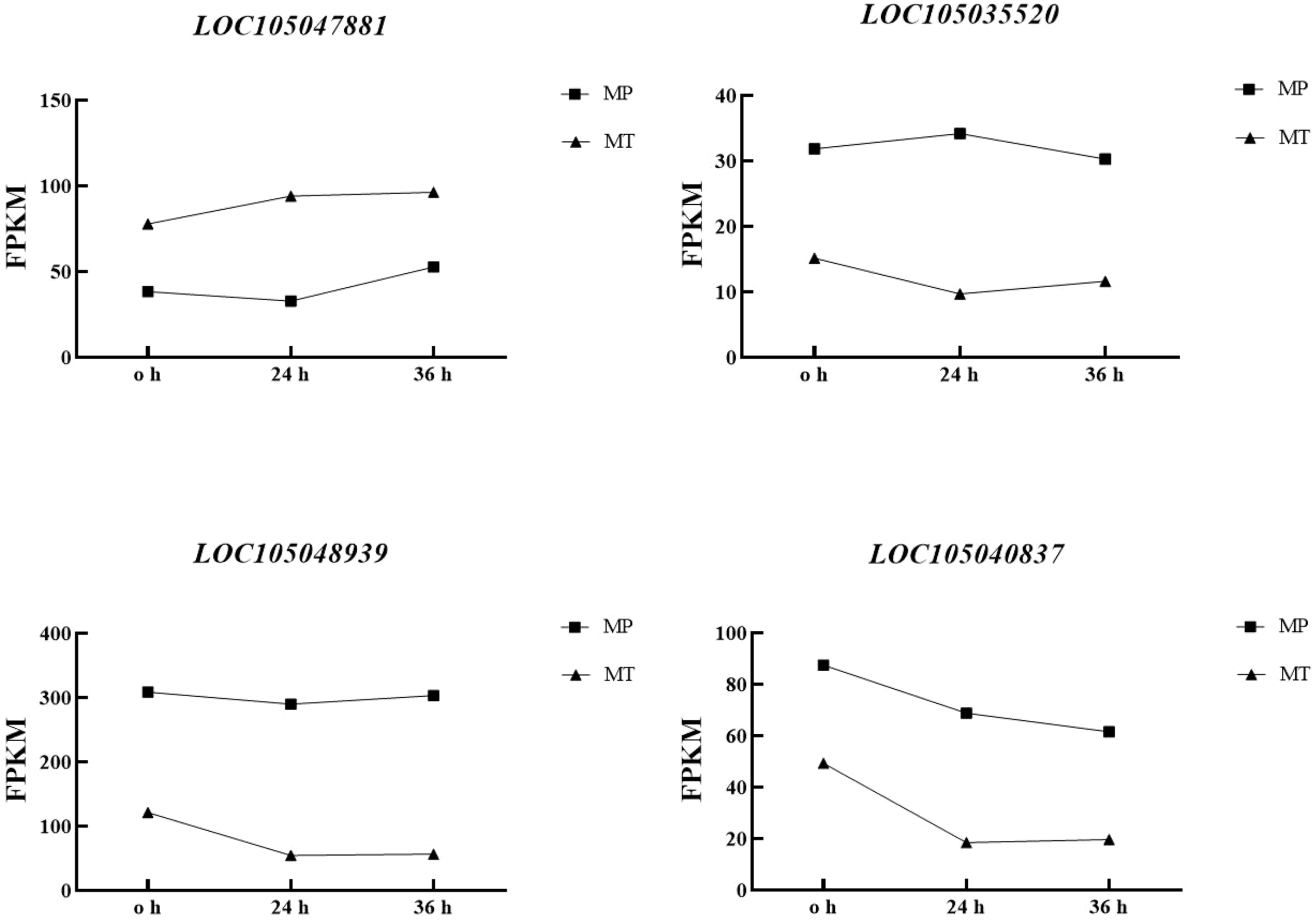
Dynamic changes of key enzyme gene expression in MT and MP oil palm species postharvest.

Correlation analysis of the dynamic changes in significant differential metabolite content (**Table 1**) and the changes in the expression of the four enzyme genes (**Figure 6**). **Table 4** showed that the contents of the four main free fatty acids in oil palm fruits were significantly correlated with the expression of the four key enzyme genes.*FATB* is positively correlated with Myristic acid, Stearic acid and Palmitic acid content, but not with Palmitoleic acid content. These results indicate that *FATB* promotes the synthesis of Myristic acid, Stearic acid and Palmitic acid, and may inhibit the synthesis of Palmitoleic acid. *SDR*, *FATA* and *MFP* are negatively correlated with Myristic acid, Stearic acid and Palmitic acid contents, while Palmitoleic acid content is positively correlated or has no high correlation. These results indicate that these three enzyme genes may inhibit the synthesis of Myristic acid, Stearic acid and Palmitic acid, and may promote the synthesis of Palmitoleic acid. From the dynamic changes in the expression levels of four key enzyme genes and proteins (**Figure 6**), it can be seen that the expression levels of *FATA* gene and *MFP* protein have the same trend in MT and MP, with higher expression levels in MP. *FATB* changes in MT and MP are inconsistent. It decreases first and then increases in MP, while it continues to increase in MT. The change of *SDR* gene expression was the opposite in the two shell types, so the difference between the two shell types was obvious.

### 3.5 QRT-PCR of the transcriptomic data

**Figure 7:**
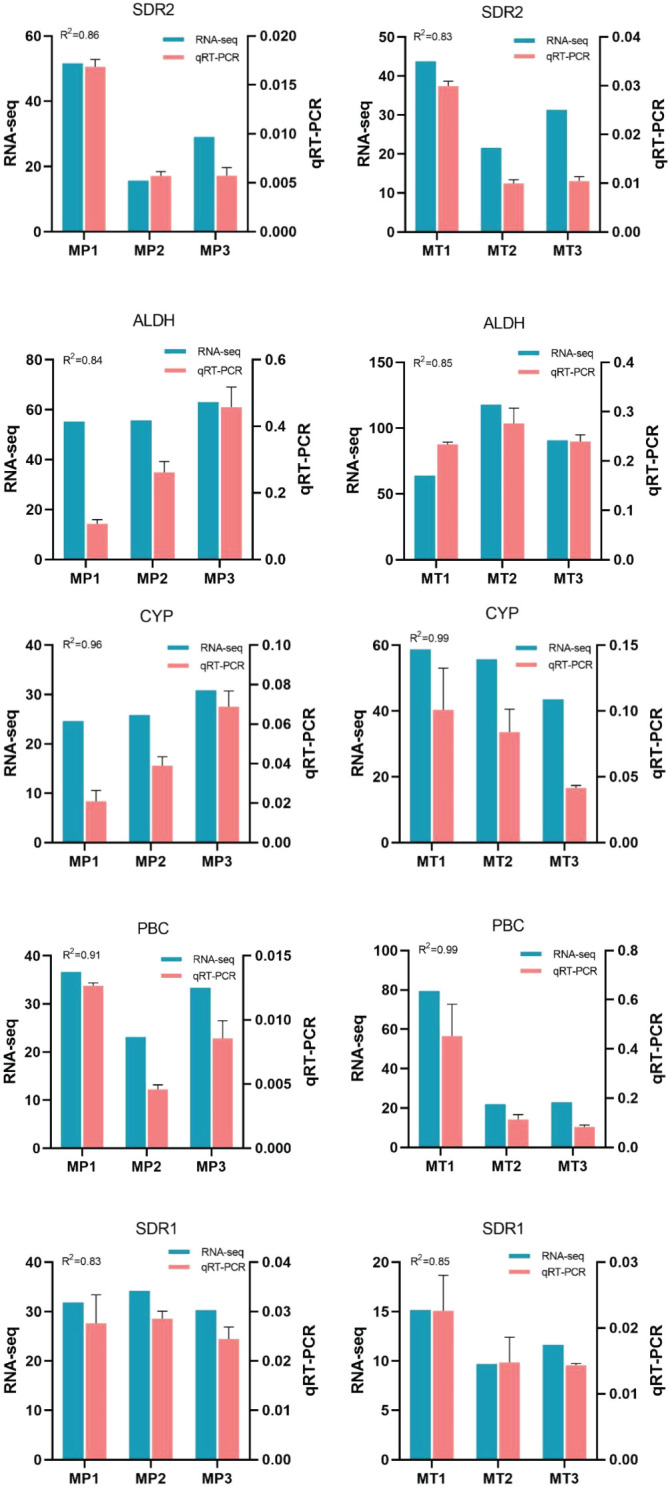
Relative expression levels of 5 selected genes during fruits postharvest rancidity stages (MP1, MP2, MP3, MT1, MT2, and MT3). The 2-ΔΔCt method was used to determine the relative expression levels of genes. The statistical differences were analyzed by ANOVA based on Duncan’s multiple test (P<0.05). Different letters indicate significant differences in the relative expression level and FPKM values.

To verify the accuracy of RNA-Seq expression profile sequencing, 5 differentially expressed genes were randomly selected from related metabolic pathways and analyzed by qRT-PCR (**Figure 7**). The results showed that the trend of qRT-PCR was consistent with that of RNA-Seq, and the coefficient of determination (R^2^) was greater than 0.8.

## 4 Discussion

PO provides 45% of the world’s edible oil and is the preferred vegetable oil to relieve the pressure on global edible oil (Li et al., 2016). Free fatty acids will produce harmful substances after oxidative rancidity. Therefore, deep analysis of the related mechanisms of fatty acid rancidity in oil palm fruits is of great importance for inhibiting fatty acid rancidity, ensuring oil safety and extending the storage life of oil foods. In this study, LC-MS/MS metabolomics technology was used to analyze the free fatty acid metabolites in the fruits of MT and MP at three postharvest times. The results showed that the total free fatty acid content of the two shell types of oil palm fruits was significantly different, and the total free fatty acid content of MT was higher.

*FAT* in plants can be divided into *FATA* and *FATB* according to differences in amino acid sequences (Jones et al., 1995). Different FAT have different catalytic activities on different substrates. *FATA* is an important factor affecting oil accumulation. Most *FATA* genes in most plants have similar functions and have catalytic activity on C18: X-ACP (Kaczmarzyk et al., 2016; Tan and Lee, 2017). However, Moreno-Pérez et al. (2012) found that silencing *AtFATA* gene in *Arabidopsis* significantly increased the contents of Linolenic acid (C18:3) and erucic acid (C22:1) in *Arabidopsis* seeds, which provided a reference for the results of this study. The *FATA* gene was found to be highly expressed in MT and low expressed in MP, and it was found to be negatively correlated with Myristic acid, Stearic acid, and Palmitic acid content and positively correlated with palmitoleic acid content in this study. Combined with the dynamic changes of free fatty acid content in the metabolome, it can be inferred that the expression of *FATA* may inhibit the synthesis of Myristic acid, Stearic acid and Palmitic acid in MT and MP, and promote the synthesis of Palmitoleic acid. The *FATB* gene family mainly tends to use saturated fatty acids as substrates, especially C16:0-ACP, which has the highest catalytic activity, but some *FATBs* have been reported to have specific activity for unsaturated fatty acids (Salas and Ohlrogge, 2002; Sánchez-García et al., 2010). *FATB* overexpression in *tobacco* was found to lead to a 1.9-fold increase in oil content in *Arabidopsis* compared with controls (El Tahchy et al., 2017). Xiong et al. (2021) found that the oil content of yeast transformers of two *FATB* genes was significantly increased in the cloning and functional preliminary analysis of five fatty acyl-ACP thioesterase genes in *Brassica napus* L. and further transformed these two genes into *Arabidopsis thaliana*, finding that the content of saturated fatty acids in *Arabidopsis thaliana* was significantly increased. The *FATB* gene was found to be highly expressed low in MP and low expressed in MT, and it was found to be positively correlated with myristic acid, stearic acid, and palmitic acid content and negatively correlated with palmitoleic acid content in this study. Combined with the dynamic changes of free fatty acid content in the metabolome, it can be inferred that the expression of *FATB* may promote the synthesis of Myristic acid, Stearic acid and Palmitic acid in MP and MT, and inhibit the synthesis of Palmitoleic acid. The above results showed that *FATA* and *FATB* showed opposite characteristics in the difference of fatty acid rancidity between MT and MP of oil palm. Therefore, *FATA* and *FATB* genes play an important role in differentiating the fatty acid rancidity of the two shell types of oil palm.

*SDR* is used in *Arabidopsis thaliana*, *Populus*, *Vitis vinifera*, *Glycine max*, *Oryza sativa*, *Zea mays* and *Sorghum bicolor* (Labesse et al., 1994; Kramm et al., 2012; Xie et al., 2022). *SDRs* have various functions in plants, some are involved in primary metabolic pathways, such as fatty acid biosynthesis, chlorophyll synthesis and degradation, while other *SDRs* are mainly involved in secondary metabolisms, such as steroid, alkaloid, terpenoid synthesis, plant aroma and phytohormone synthesis, etc. (Stavrinides et al., 2018; Zhang et al., 2020). The *SDR* family includes -ketoacyl-ACP reductase (BKR) and enoyl-ACP reductase (ENR) in *de novo* fatty acid synthesis, which are involved in NADPH-dependent reduction reactions in fatty acid chain extension (Tonfack et al., 2011). Mou et al. (2000) found that deletion of ENR in *Arabidopsis* leads to a reduction of fatty acid content. Therefore, *SDR* proteins plays an important role in fatty acid synthesis. Wang et al. (2022) found in the functional study of apple short-chain dehydrogenase *MdSDR* that after transient overexpression of *MdSDR*. The genes acetylCoA carboxylase (MdACCase), -ketoacyl-ACP synthase (MdKAS), -ketoacyl-ACP reductase (MdKAR), and enylacyl-ACP reductase (MdENR) were significantly up-regulated in apples. These results indicate that Md*SDR* could participate in the regulation of plant fatty acid biosynthesis. In this study, according to the pathway shown on KEGG, *SDR* is negatively correlated with Myristic acid, Stearic acid and Palmitic acid content, but not with Palmitoleic acid content. Therefore, it is speculated that *SDR* may inhibit the synthesis of Myristic acid, Stearic acid and Palmitic acid and promote the synthesis of Palmitoleic acid in the rancidation process of oil palm fatty acids. However, *SDR* has a high expression level in MP and a low expression level in MT, which is the key enzyme gene responsible for the rout difference between MT and MP oil palm.

The β-oxidation of fatty acids in peroxisomes can be divided into two metabolic pathways based on *MFP* substrate specificity: the straight-chain fatty acid oxidation pathway and the branched-chain fatty acid oxidation pathway (Graham and Eastmond, 2002). Wei (2009) found that *MFP* protein was involved in regulating the biodecomposition of fatty acids and their derivatives in the study of EST-SSR development in sesame, genetic map construction and gene cloning related to fatty acid metabolism in cotton. Wang et al. (2016) investigated the cloning and prokaryotic expression of the multifunctional protein gene of fatty acid β-oxidation in *Camellia oleifera* and found that *CoMFP* plays a multifunctional enzyme role of hydration and dehydrogenation in fatty acid β-oxidation in *camellia oleifera*. This study found that *MFP* protein is negatively correlated with the contents of Myristic acid, Stearic acid and Palmitic acid in the process of fatty acid rancor of MT and MP, and that its expression is higher in MP. These results highlighted that the expression of *MFP* protein has a stronger degradation effect on Myristic acid, Stearic acid and Palmitic acid in MP, and therefore, the contents of these three fatty acids are lower in MP. Consequently, it is speculated that *MFP* protein may be the key enzyme gene for the difference in fatty acid rancidity between MT and MP in oil palm.

## 5 Conclusions

By combining metabolomics and transcriptomics analysis, we found that *FATA*, *SDR* and *MFP* enzyme genes and enzyme proteins were significantly negatively correlated with Myristic acid, Stearic acid and Palmitic acid in the rancidation process of oil palm fruits. These results indicate that the expression of these enzyme genes and enzyme proteins could promote the degradation of free fatty acids in the rancidity process of oil palm fruits. The expression of the *FATB* gene is positively correlated with Myristic acid, Stearic acid and Palmitic acid in oil palm fruit rancidity, indicating that the expression of *FATB* gene promotes the synthesis of free fatty acids in oil palm fruit rancidity. Combined with gene expression, *FATA* gene and *MFP* protein expression showed the same trend in MT and MP, with higher expression levels in MP. The changes to *FATB* in MT and MP were inconsistent. The expression of *FATB* in MP decreased first and then increased, while the expression of *FATB* in MT continued to increase. The changes in *SDR* gene expression were opposite in the two shell types. The above results clearly show the differences between the two shell types. In addition, MP2-vs-MT2 contained 12 free fatty acids and 4947 significant differentially expressed genes. As a result, it was found that the most obvious time of fatty acid rancidity difference between MT and MP shell types was 24 hours postharvest. The aim of this study was to compare the molecular regulation mechanism of free fatty acid metabolism in oil palm shell types with other shell types, in order to provide a theoretical basis for further genetic improvement using molecular biotechnology and promote the development of the oil palm industry.

## Data availability statement

The data presented in the study are deposited in the NCBI repository, accession number PRJNA932459.

## Author contributions

SZ, WZ, XF, MF, LW, AZ and CY conducted the experiments and performed the data analysis. HC organized and supervised the overall project. SZ and WZ wrote the manuscript. JM and RQ edited the manuscript. All authors contributed to the article and approved the submitted version.

## References

[B1] Ahmed M. Pickova J. Ahmad T. Liaquat M. Farid A. Jahangir M. (2016). Oxidation of lipids in foods. Sarhad J. Agric. 32 (3), 230–238. doi: 10.17582/journal.sja/2016.32.3.230.238

[B2] Boateng L. Ansong R. Owusu W. B. Steiner-Asiedu M. (2016). Coconut oil and palm oil's role in nutrition, health and national development: A review. Ghana Med. J. 50 (3), 189–196. doi: 10.4314/gmj.v50i3.11 27752194 PMC5044790

[B3] Clegg A. J. (1973). Composition and related nutritional and organoleptic aspects of palm oil. J. Am. Oil Chemists' Society. 50 (8), 321–324. doi: 10.1007/BF02641365 4785178

[B4] Damayant S. Andry S. Khairurrijal. Kartasasmita R. E. (2014). Isolation of β-carotene from palm (*Elaeis guineensis jacq.*) oil using transesterification-adsorption-desorption method and its characterization. J. Appl. Sci. 14, 2615–2621. doi: 10.3923/jas.2014.2615.2621

[B5] Dieuaide-Noubhani M. Novikov D. Baumgart E. Vanhooren J. C. Fransen M. Goethals M.. (1996). Further characterization of the peroxisomal 3-hydroxy acyl-CoA dehydrogenases from rat liver. relationship between the different dehydrogenases and evidence that fatty acids and the C27 bile acids di- and tri-hydroxycoprostanic acids are metabolized by separate multifunctional proteins. Eur. J. Biochem. 15;240 (3), 660–666. doi: 10.1111/j.1432-1033.1996.0660h.x 8856068

[B6] Dieuaide-Noubhani M. Novikov D. Vandekerckhove J. Veldhoven P. P. Mannaerts G. P. (1997). Identification and characterization of the 2-enoyl-CoA hydratases involved in peroxisomal β-oxidation in rat liver. Biochem. J. 321 (Pt1), 253–259. doi: 10.1042/bj3210253 9003427 PMC1218062

[B7] Dussert S. Guerin C. Andersson M. Joët T. Tranbarger T. J. Pizot M.. (2013). Comparative transcriptome analysis of three oil palm fruit and seed tissues that differ in oil content and fatty acid composition. Plant Physiol. 162 (3), 1337–1358. doi: 10.1104/pp.113.220525 23735505 PMC3707537

[B8] El Tahchy A. Reynolds K. B. Petrie J. R. Singh S. P. Vanhercke T. (2017). Thioesterase overexpression in nicotiana benthamiana leaf increases the fatty acid flux into triacylgycerol. FEBS Lett. 591 (2), 448–456. doi: 10.1002/1873-3468.12539 28024101

[B9] Gong W. Lv L. Wang W. Huang L. (2020). Gene cloning and sequence analysis of *MdSDR* in *SDRs* family from Fuji apple. Mol. Plant Breed. 18 (14), 4597–4604.

[B10] Graham IA Eastmond PJ. (2002). Pathways of straight and branched chain fatty acid catabolism in higher plants. Prog Lipid Res. 41 (2), 156–181. 11755682 10.1016/s0163-7827(01)00022-4

[B11] Jones A. Davies H. M. Voelker T. A. (1995). Palmitoyl-acyl carrier protein (ACP) thioesterase and the evolutionary origin of plant acyl-ACP thioesterases. Plant Cell. 7 (3), 359–371. doi: 10.1105/tpc.7.3.359 7734968 PMC160788

[B12] Kaczmarzyk D. Hudson E. P. Fulda M. (2016). *Arabidopsis* acyl-acylcarrier protein synthetase AAE15 with medium chain fatty acid specificity is functional in cyanobacteria. AMB Express. 6 (1), 1–9. doi: 10.1186/s13568-016-0178-z 26797881 PMC4722043

[B13] Kadandale S. Marten R. Smith R. (2019). The palm oil industry and noncommunicable diseases. B World Health Organ. 97 (2), 118–128. doi: 10.2471/BLT.18.220434 PMC6357563 30728618

[B14] Kramm A. Kisiela M. Schulz R. Maser E. (2012). Short-chain dehydrogenases/reductases in *cyanobacteria* . FEBS J. 279 (6), 1030–1043. doi: 10.1111/j.1742-4658.2012.08494.x 22251568

[B15] Labesse G. Vidal-Cros A. Chomilier J. Gaudry M. Mornon J. P. (1994). Structural comparisons lead to the definition of a new superfamily of NAD(P)(H)-accepting oxidoreductases: The single-domain reductases/epimerases/dehydrogenases(the 'RED' family). Bioche J. 304 (Pt 1), 95–99. doi: 10.1042/bj3040095 PMC1137457 7998963

[B16] Li Y. Bao H. Lai X. Wu J. (1997). Research on the oxidation and antioxidation of edible oils and fats. China Food additives. 04), 5–9.

[B17] Li J. Wang Y. Yang Y. (2016). Comparison of fatty acid component between palm oil and common edible oils. J. South. Agriculture. 47 (12), 2124–2128.

[B18] Liu J. (2021). Detection methods of free fatty acids and its clinical application value. Chin. J. Med. Device. 34 (19), 195–196.

[B19] Manorama R. Rukmini C. (1991). Nutritional evaluation of crude palm oil in rats. Am. J. Clin. Nutr. 53 (4), 1031S–1033S. doi: 10.1093/ajcn/53.4.1031S 2012012

[B20] Mayer K. M. Shanklin J. (2007). Identification of amino acid residues involved in substrate specificity of plant acyl-ACP thioesterases using a bioinformatics-guided approach. BMC Plant Biol. 7, 1. doi: 10.1186/1471-2229-7-1 17201914 PMC1770913

[B21] Mba O. I. Dumont M. J. Ngadi M. (2015). Palm oil: Processing, characterization and utilization in the food industry -a review. Food Biosci. 10, 26–41. doi: 10.1016/j.fbio.2015.01.003

[B22] Moreno-Pérez A. J. Venegas-Calerón M. Vaistij F. E. Salas J. J. Larson T. R. Garcés R.. (2012). Reduced expression of *FatA* thioesterases in *Arabidopsis* affects the oil content and fatty acid composition of the seeds. Planta. 235 (3), 629–639. doi: 10.1007/s00425-011-1534-5 22002626

[B23] Mou Z. He Y. Dai Y. Liu X. Li J. (2000). Deficiency in fatty acid synthase leads to premature cell death and dramatic alterations in plant morphology. Plant Cell 12 (3), 405–417. doi: 10.1105/tpc.12.3.405 10715326 PMC139840

[B24] Murphy D. J. (2007). Future prospects for oil palm in the 21st century: Biological and related challenges. Eur. J. Lipid Sci. Technol. 109 (4), 296–306. doi: 10.1002/ejlt.200600229

[B25] Novikov D. K. Vanhove G. F. Carchon H. Asselberghs S. Eyssen H. J. Veldhoven. P. P.. (1994). Peroxisomal β-oxidation: Purification of four novel 3-hydroxyacyl-coA dehydrogenases from rat liver peroxisomes. J. Biol. Chem. 269 (43), 27125–27135. doi: 10.1016/S0021-9258(18)47134-7 7929456

[B26] Olafisoye O. B. Oguntibeju O. O. Osibote O. A. (2015). Trace elements and radionuclides in palm oil, soil, water, and leaves from oil palm plantations: A review. Crit. Rev. Food Sci. Nutr. 57 (7), 1295–1315. doi: 10.1080/10408398.2014.886032 25875248

[B27] Salas J. J. Ohlrogge J. B. (2002). Characterization of substrate specificity of plant *FatA* and *FatB* acyl-ACP thioesterases. Arch. Biochem. Biophys. 403 (1), 25–34. doi: 10.1016/S0003-9861(02)00017-6 12061798

[B28] Sánchez-García A. Moreno-Pérez A. J. Muro-Pastor A. M. Salas J. J. Garcés R. Martínez-Force E. (2010). Acyl-ACP thioesterases from castor (Ricinus communis l.): An enzymatic system appropriate for high rates of oil synthesis and accumulation. Phytochemistry. 71 (8-9), 860–869. doi: 10.1016/j.phytochem.2010.03.015 20382402

[B29] Stavrinides A. K. Tatsis E. C. Dang T. T. Caputi L. Stevenson C. E. M. Lawson D. M.. (2018). Discovery of a short-chain dehydrogenase from catharanthus roseus that produces a new monoterpene indole alkaloid. Chembiochem. 19 (9), 940–948. doi: 10.1002/cbic.201700621 29424954 PMC6003104

[B30] Sun L. Dong X. Liu Y. Jiang A. Wang F. Weng X. (1998). Autoxidative mechanism of lipids. China Oils Fats. 05), 56–57.

[B31] Tan K. Lee Y. K. (2017). Expression of the heterologous dunaliella tertiolecta fatty acyl-ACP thioesterase leads to increased lipid production in chlamyd omonas reinhardtii. J. Biotechnol. 247 (Complete), 60–67. doi: 10.1016/j.jbiotec.2017.03.004 28279815

[B32] Tonfack L. B. Moummou H. Youmbi E. Benichou M. Pech J. C. van der Rest B. (2011). The plant *SDR* superfamily: Involvement in primary and secondary metabolism. Curr. Topics Plant Biol. 12, 41–53.

[B33] Wang X. (2004). Oxidation of oil and test methods of oxidative stability of oil. J. Dezhou University. 20 (6), 46–50.

[B34] Wang J. Tan X. Zeng Y. Long H. Chen H. Liu K.. (2016). Cloning and prokaryotic expression of a fatty acid b-oxidation multifunctional protein gene from c*amellia oleifera* . Mol. Plant Breeding. 14 (06), 1410–1420.

[B35] Wang Q. Tan C. Zhang L. Cui B. (2014). The factors influencing oxidative rancidity of edible oil and its prevention. Jiangsu Condiment Subsidiary Food. 03), 9–12.

[B36] Wang S. Xiao K. Wang W. Huang L. (2022). Function exploration of apple short-chain Dehydrogenases/Redutase in response to. Valsa mali. J. Nucl. Agric. Sci. 36 (11), 2158–2165.

[B37] Wei L. (2009). EST-SSR Development Genetic mapping construction in sesame and cloning of genes related with fatty acid metabolism in cotton.

[B38] Xia Q. Li R. Tang M. Wang W. Lei X. Zhao S. (2011). Fatty acid composition and antioxidant property of the oils from oil palm produced in wenchang city, hainan. Chin. J. Trop. Crops. 32 (05), 906–910.

[B39] Xie X. Chen Q. Wang L. P. Wang Q. (2022). Cloning and functional analysis of rice short-chain dehydrogenase genes. Mol. Plant Breeding. 20 (01), 7–14.

[B40] Xiong T. Chen Z. Zhang Z. Chen H. Yuan Y. Xiong X.. (2021). Gene isolation and characterization of 5 fatty acyl-ACP thioesterase in *Brassica napus* . Chin. J. Oil Crop Sci. 43 (02), 212–218.

[B41] Xiong H. Li R. Li X. Fan H. Ma Z. (2009). Investigation, analysis and the advice of palm industry in China. Chin. Agricult Sci. Bul-let. 25, 114–117.

[B42] Yang C. Li Z. Rong R. (2010). Mechanism and prevention of oxidation in plant oils. Acad. timeical Farm Products Process. 12), 85–88.

[B43] Zhang Y. Qin Z. Wang J. Wang X. (2020). Short-chain dehydrogenase research advancein secondary metabolism of plant. Nanfang Forestry Science. 48 (05), 62–67.

