# Supplementary material for: Differential analysis of transcriptomic and metabolomic of free fatty acid rancidity process in oil palm (Elaeis guineensis) fruits of different husk types
Source: Front Plant Sci. 2023 Mar 8;14:1132024. doi: 10.3389/fpls.2023.1132024 (PMC10030942; doi:10.3389/fpls.2023.1132024)
Supplement: Supplementary file 1 [file DataSheet_1.doc]

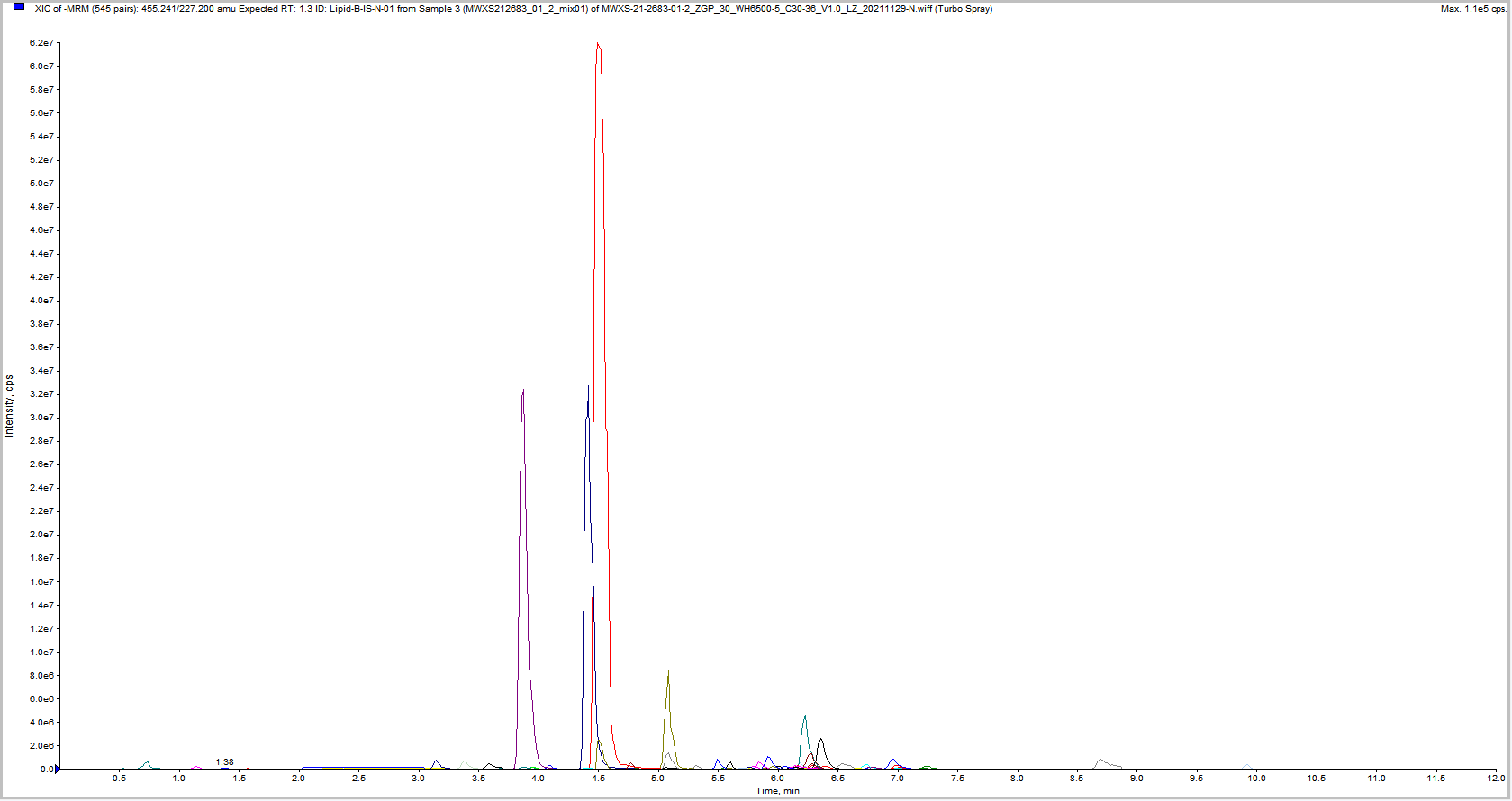

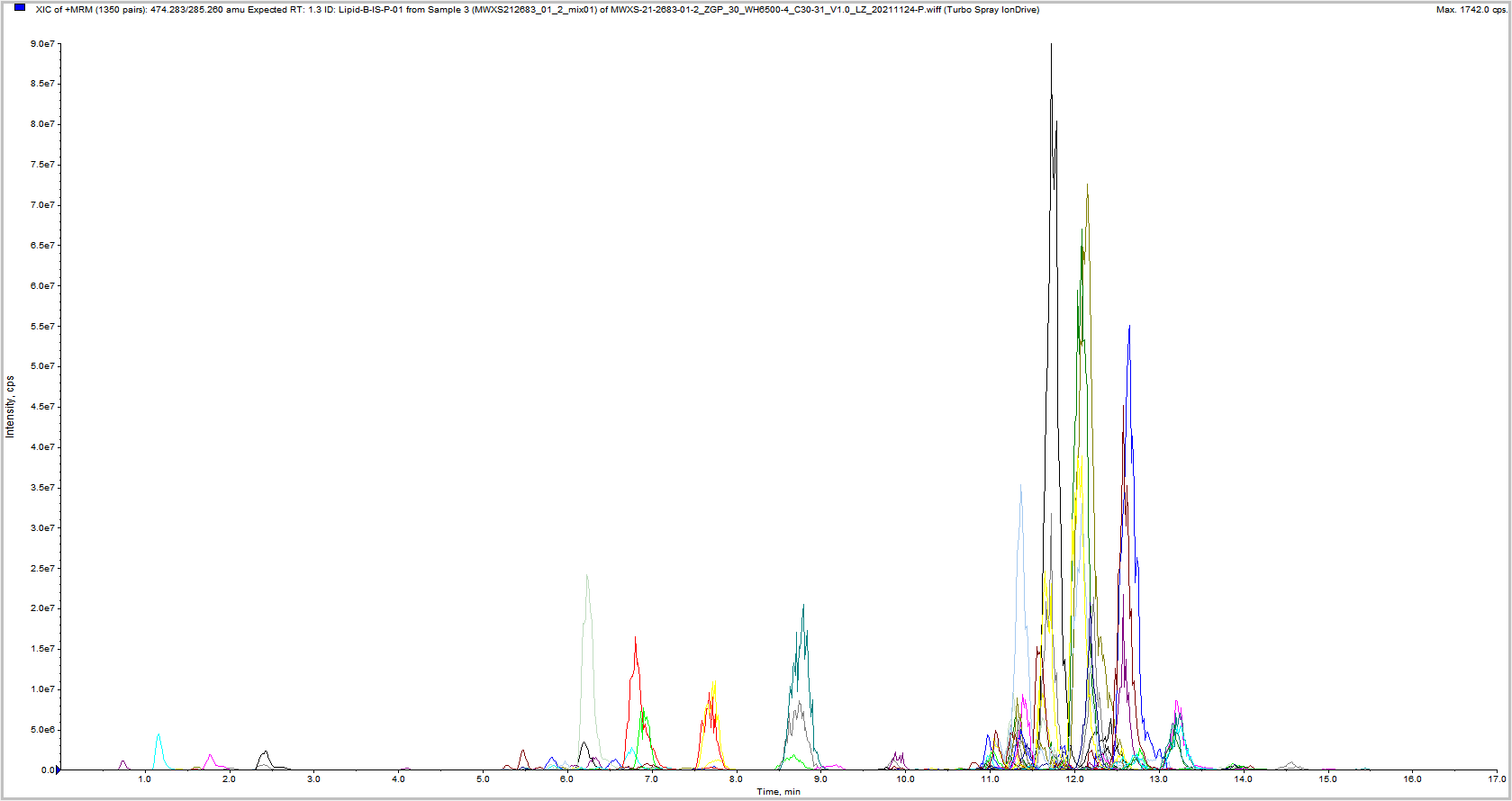

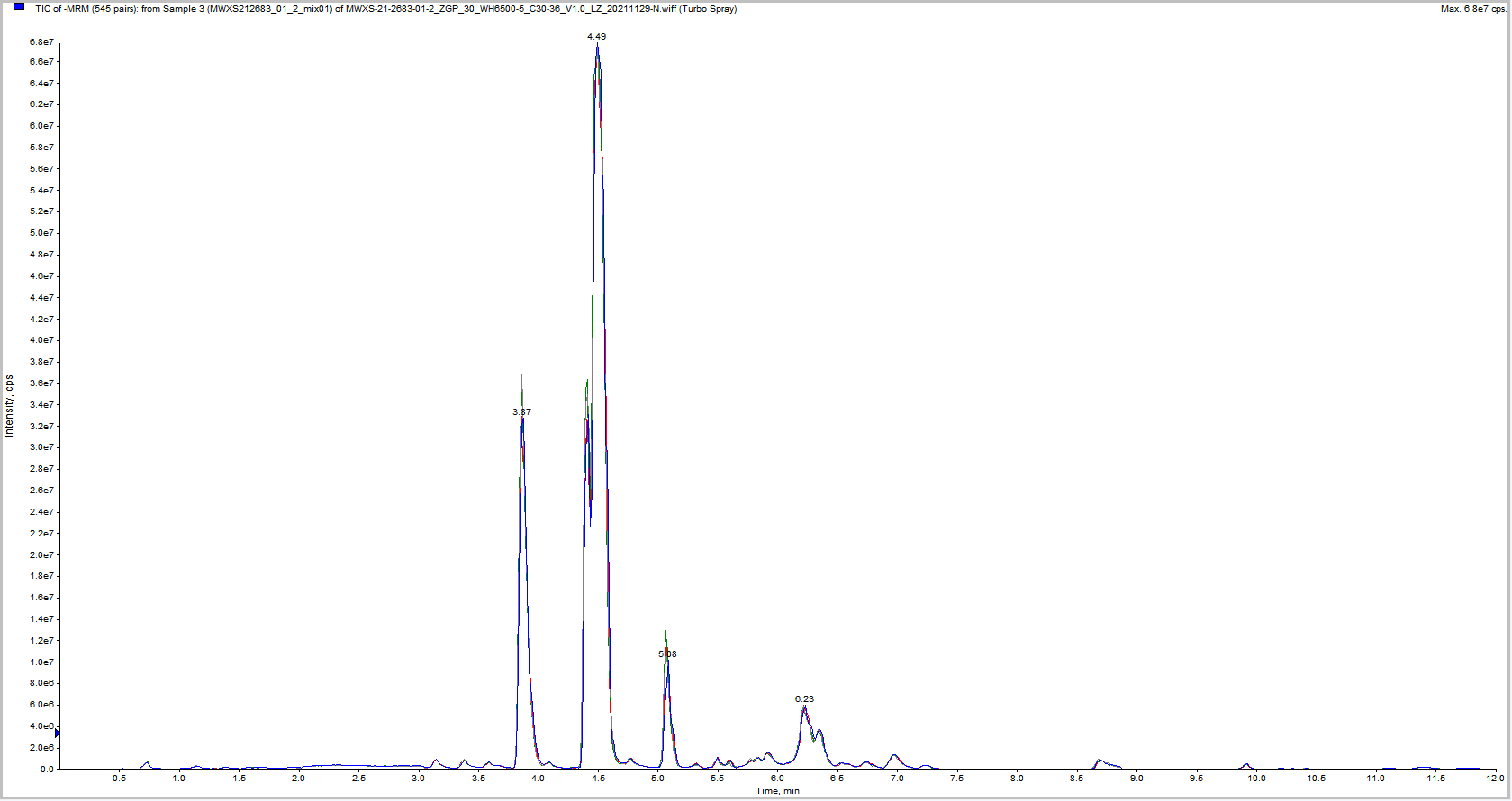

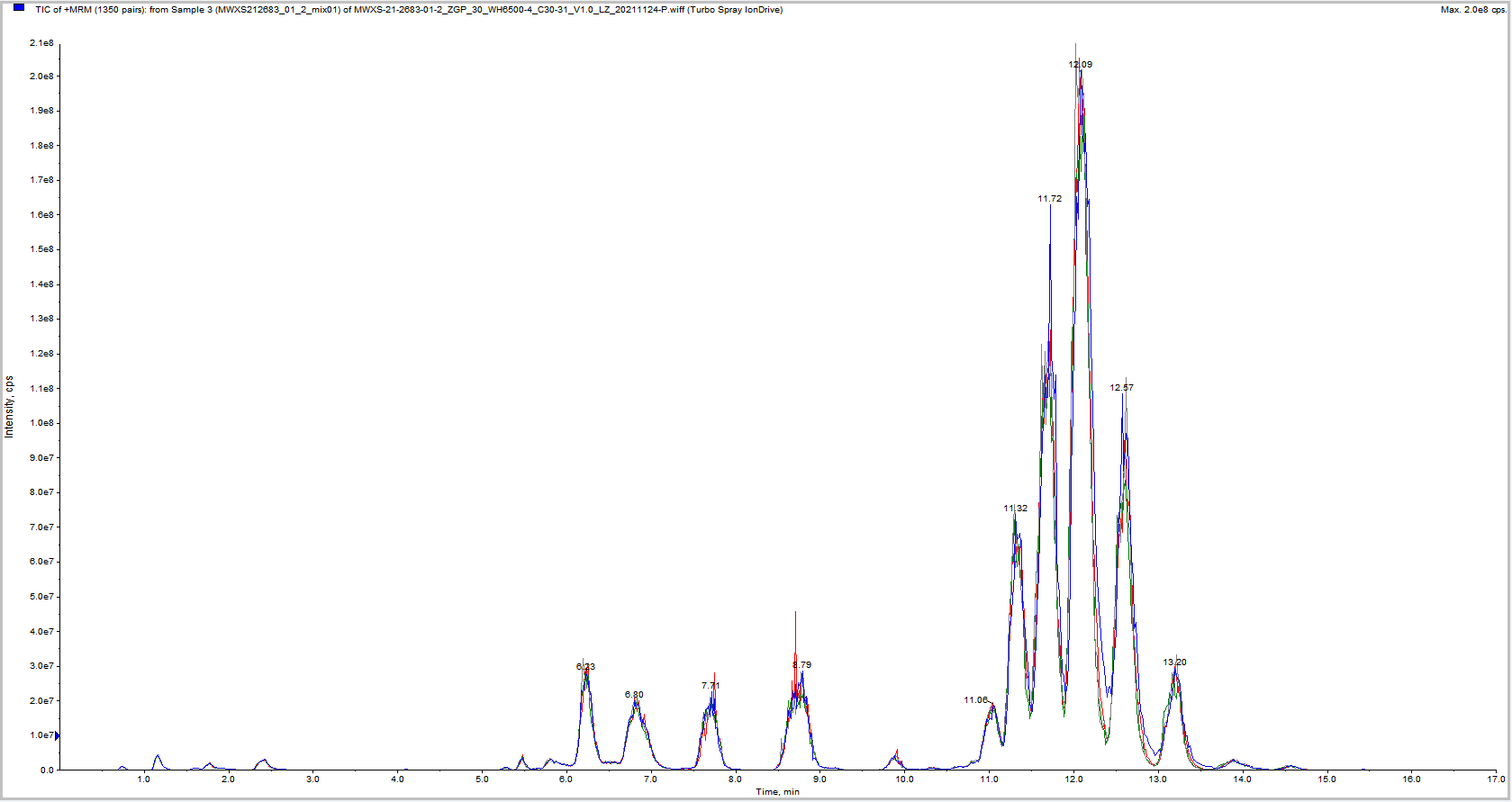

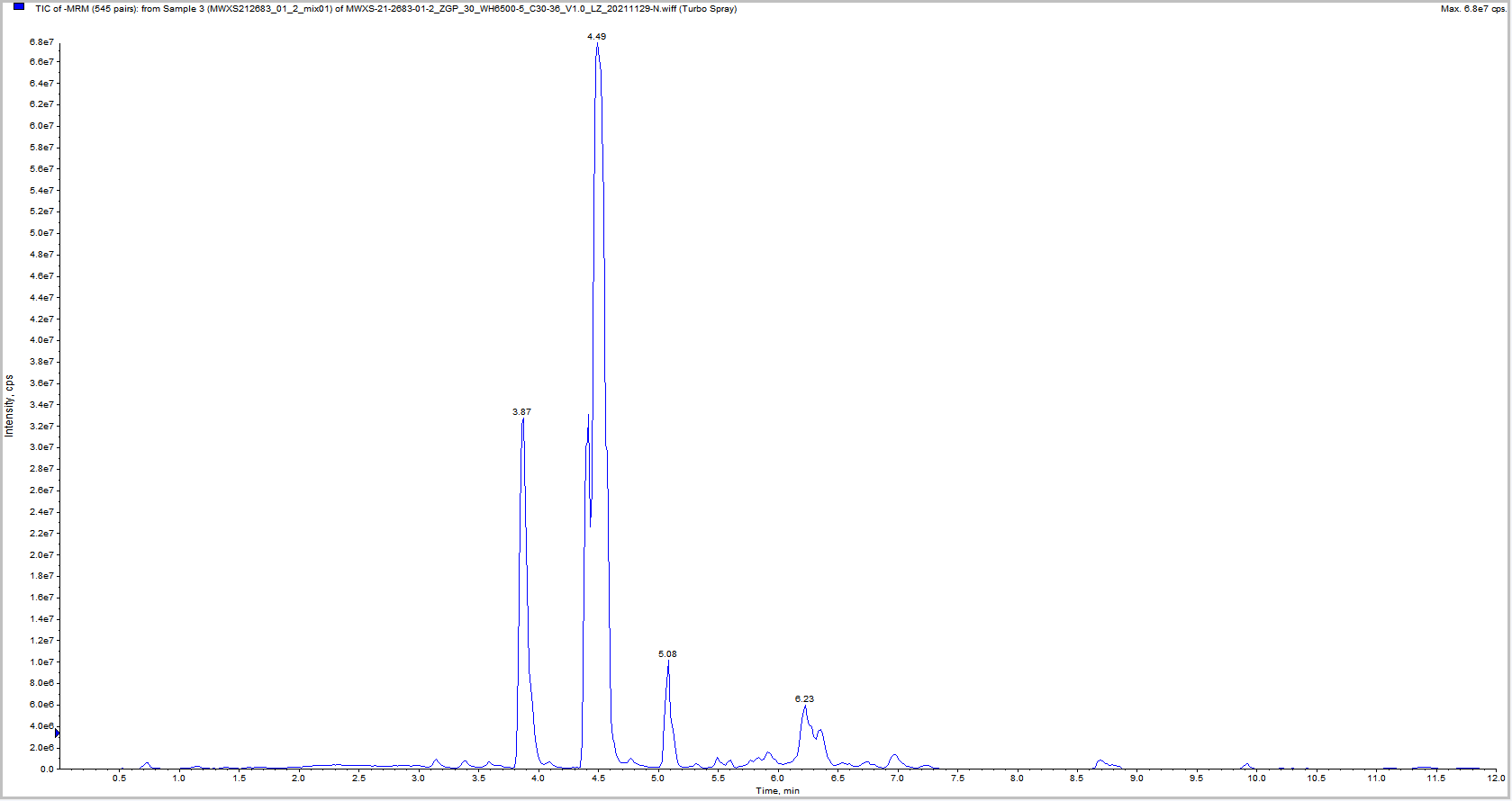

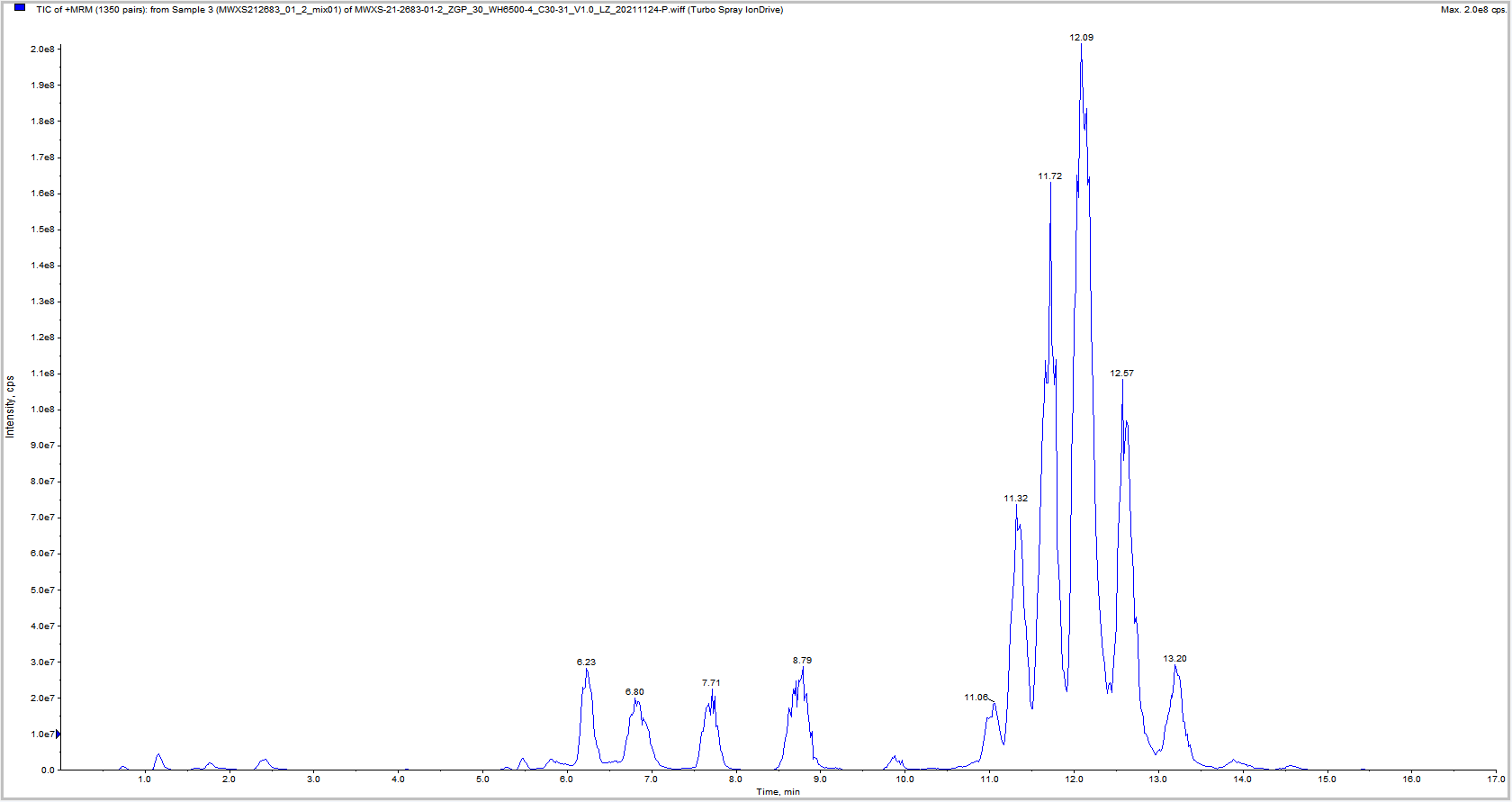


**Supplementary Figure 1: MRM model**


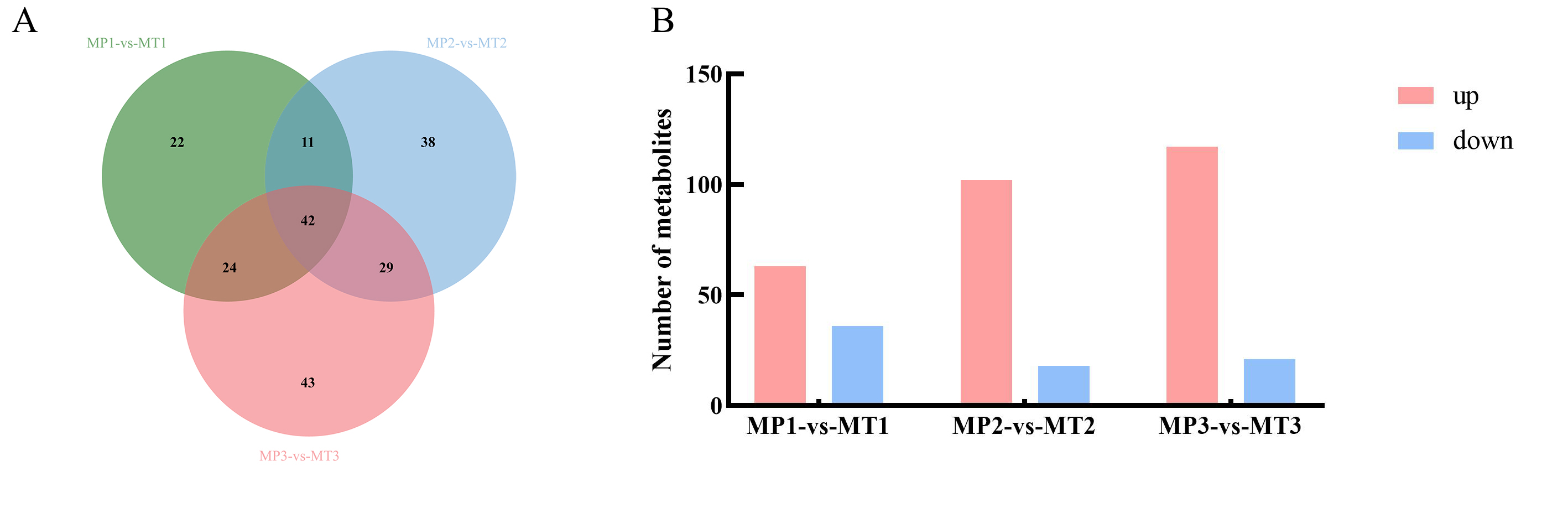
**Supplementary Figure 2: Statistics of MP and MT oil palm significant differential metabolite**

**Note:** A: Venn diagram of lipid metabolism with a significant difference; B: Histogram of lipid metabolism with a significant difference

**Supplementary Table 1: Mass of RNA-seq**

| Sample | Raw Reads | Clean Reads | Clean Base(G) | Error Rate(%) | Q20(%) | Q30(%) | GC Content(%) |
| --- | --- | --- | --- | --- | --- | --- | --- |
| MP1-1 | 48740904 | 46390726 | 6.96 | 0.03 | 97.76 | 93.52 | 48.97 |
| MP1-2 | 47079366 | 45077920 | 6.76 | 0.03 | 97.65 | 93.29 | 48.99 |
| MP1-3 | 57890096 | 56348144 | 8.45 | 0.03 | 97.39 | 92.85 | 48.47 |
| MP2-1 | 49030854 | 46624048 | 6.99 | 0.03 | 97.54 | 92.98 | 49.1 |
| MP2-2 | 48064768 | 46102110 | 6.92 | 0.03 | 97.55 | 93.05 | 49.08 |
| MP2-3 | 48229894 | 45298346 | 6.79 | 0.03 | 97.56 | 93.03 | 48.97 |
| MP3-1 | 47806494 | 46034050 | 6.91 | 0.03 | 97.56 | 93.06 | 49.32 |
| MP3-2 | 47753064 | 45613090 | 6.84 | 0.03 | 97.76 | 93.47 | 48.84 |
| MP3-3 | 47550062 | 45337056 | 6.8 | 0.03 | 97.73 | 93.46 | 49.67 |
| MT1-1 | 48145634 | 44847734 | 6.73 | 0.03 | 97.67 | 93.32 | 48.73 |
| MT1-2 | 46633640 | 43299316 | 6.49 | 0.03 | 97.18 | 92.16 | 48.83 |
| MT1-3 | 47406742 | 43203442 | 6.48 | 0.03 | 97.86 | 93.95 | 48.56 |
| MT2-1 | 48888356 | 45481814 | 6.82 | 0.03 | 97.69 | 93.35 | 48.82 |
| MT2-2 | 46807928 | 43234536 | 6.49 | 0.03 | 97.74 | 93.46 | 49.25 |
| MT2-3 | 48223554 | 44390124 | 6.66 | 0.03 | 97.74 | 93.45 | 49.12 |
| MT3-1 | 47706726 | 43081970 | 6.46 | 0.03 | 97.93 | 94.08 | 48.84 |
| MT3-2 | 47161986 | 42357142 | 6.35 | 0.02 | 98.03 | 94.3 | 48.54 |
| MT3-3 | 48203562 | 42683588 | 6.4 | 0.03 | 97.96 | 94.18 | 48.86 |
